# Hemodynamic cortical ripples through cyclicity analysis

**DOI:** 10.1162/netn_a_00392

**Published:** 2024-12-10

**Authors:** Ivan Abraham, Somayeh Shahsavarani, Benjamin Zimmerman, Fatima T. Husain, Yuliy Baryshnikov

**Affiliations:** Coordinated Science Laboratory, University of Illinois, Urbana-Champaign, Urbana, USA; Department of Audiology, San Jose State University, San Jose, USA; Helfgott Institute, National University of Natural Medicine, Portland, USA; Beckman Institute for Advanced Science & Technology, University of Illinois, Urbana-Champaign, USA; Department of Speech & Hearing Science, University of Illinois, Urbana-Champaign, Urbana, USA; Department of Mathematics, University of Illinois, Urbana-Champaign, USA

**Keywords:** Functional MRI, Dynamic networks, Cortical waves, Cyclicity analysis, Iterated integrals

## Abstract

A fine-grained understanding of dynamics in cortical networks is crucial to unpacking brain function. Resting-state functional magnetic resonance imaging (fMRI) gives rise to time series recordings of the activity of different brain regions, which are aperiodic and lack a base frequency. Cyclicity analysis, a novel technique robust under time reparametrizations, is effective in recovering the temporal ordering of such time series, collectively considered components of a multidimensional trajectory. Here, we extend this analytical method for characterizing the dynamic interaction between distant brain regions and apply it to the data from the Human Connectome Project. Our analysis detected cortical traveling waves of activity propagating along a spatial axis, resembling cortical hierarchical organization with consistent lead-lag relationships between specific brain regions in resting-state scans. In fMRI scans involving tasks, we observed short bursts of task-modulated strong temporal ordering that dominate overall lead-lag relationships between pairs of regions in the brain that align temporally with stimuli from the tasks. Our results suggest a possible role played by waves of excitation sweeping through brain regions that underlie emergent cognitive functions.

## Author Summary

While brain network studies initially used correlated signals from brain regions to infer their network structure, recent efforts have focused on the dynamic aspects of such networks. This study extends the cyclicity analysis (CA) method—a technique developed for aperiodic time series analysis—to the Human Connectome Project. Notably, CA makes no assumptions about the statistics of the data and works despite possibly nonlinear changes to the timeline of the observations. Using CA, we provide evidence for (a) the propagation of an ultraslow brain wave in the resting state and (b) the detection of directed activity between brain regions that fluctuate in the presence of tasks and stimuli, without relying on frequency domain or correlation-based analysis—a novel contribution to existing literature.

## INTRODUCTION

The brain spontaneously generates neural activity even in the absence of any sensory input, motor output, or attention-demanding cognitive tasks. Such intrinsic activity “at rest” can be indirectly measured as infraslow (<0.1 Hz) fluctuations in blood-oxygen level-dependent (BOLD) signals using functional magnetic resonance imaging (fMRI). Despite its “noisy” appearance, many studies have shown that spontaneous BOLD activity carries meaningful information, having a dynamic structure that can interact with perception, cognition, and behavior (Allen et al., 2014; Cohen, 2018; Fox & Raichle, 2007; Gonzalez-Castillo et al., 2019; Sadaghiani, Poline, Kleinschmidt, & D’Esposito, 2015; Shahsavarani et al., 2023; Shine et al., 2016; Yu et al., 2015). Conventionally, using a measure known as resting-state functional connectivity (FC), this structure has been characterized as synchronous patterns of BOLD signals in bilaterally symmetric, distant brain regions, forming time-resolved functional neural networks distributed across the brain.

An emerging corpus of work has revealed that infraslow spontaneous fluctuations of BOLD signals are organized not only spatially into neural networks but also temporally in an orderly fashion (Mitra et al., 2018; Vidaurre, Smith, & Woolrich, 2017; Zhang, Watrous, Patel, & Jacobs, 2018). That is, there are time-resolved transitions among resting-state networks, which are not random but follow a specific sequential order. It is posited that a global structure guides the switching among the neural networks, which can be explained by cortical traveling waves, sequentially activating these networks (Matsui, Murakami, & Ohki, 2016; Munn, Müller, Wainstein, & Shine, 2021; Raut et al., 2021). Previously, spontaneous and stimulus-evoked traveling waves propagating across the cortex were reported using electroencephalography (EEG) and electrocorticography (ECoG) (Massimini, Huber, Ferrarelli, Hill, & Tononi, 2004; Muller, Chavane, Reynolds, & Sejnowski, 2018). In these cases, cortical waves have been primarily noted at higher frequencies (>0.1 Hz); however, cortical traveling waves can also be identified at lower frequencies. Infraslow neuronal activity (<0.1 Hz) was first observed in the rabbit cortex’s local field potential (Aladjalova, 1957). Later, full-band EEG was used to detect this activity in humans (Vanhatalo et al., 2004). These infraslow oscillations are a fundamental feature of brain function, which correlate with higher frequency oscillations and influence behavior (Lörincz, Geall, Bao, Crunelli, & Hughes, 2009; Palva & Palva, 2012; Sihn & Kim, 2022). In addition, it has been demonstrated that these oscillations are correlated with infraslow hemodynamic activity and underlie changes in fMRI resting-state networks (Fernandez & Lüthi, 2020; Grooms et al., 2017; Hiltunen et al., 2014). Notably, using wide-field voltage-sensitive dye imaging, Chan, Mohajerani, LeDue, Wang, and Murphy (2015) have shown that the infraslow spontaneous neural fluctuations recapitulate cortical motifs at higher frequencies. Using wide-field optical calcium imaging in transgenic mice, Matsui et al. (2016) detected infraslow global traveling waves across the cortex. Importantly, they showed that the phase of these cortical traveling waves regulated the resting-state FC patterns of hemodynamic data. Using concurrently recorded intracranial EEG and fMRI measures, Mostame et al. (2022) showed that while the two measures shared similar connectome configurations, they did so at asynchronous points in time, suggesting that hemodynamic and electrophysiological signals capture distinct aspects of connectivity rather than measuring the same phenomenon.

The primary finding of this paper is the detection of traveling waves propagating across the cortex by expanding the previously presented cyclicity analysis (CA) pipeline (Zimmerman, Abraham, Schmidt, Baryshnikov, & Husain, 2018).

**We report three significant novel observations:**

- A time-resolved temporal ordering among the resting-state activity of brain regions was identified with a wave propagating along a spatial axis, resembling cortical hierarchical organization.
- The dynamics of temporal ordering in the task paradigm were primarily characterized by distinct, short-term “bursts” in contrast to gradual changes.
- The direction of wave propagation repeatedly shifts during certain tasks, a phenomenon we term as “reversals” to contrast with the unidirectional steady dynamics observed in the resting state.

Our work also uses novel methodological approaches. While CA was conceptualized and tested in the context of *cyclic* phenomena—defined as those which follow a trajectory over and over again, in a contrast with the periodic phenomena, which repeat after time shift by a well-defined interval (Baryshnikov & Schlafly, 2016)—we discovered that in the systems we analyze here, the better interpretation is that of a **ripple** sweeping through the terrain, followed by an interval of calm.

From the perspective of reparametrization-invariant data analysis, where CA belongs, the length of the calm interval is immaterial. The key difference with the cyclic situation is the fact that, at times, most of the network nodes are at rest. Exhibiting unexpected robustness, CA proved to be capable of detecting the presence and describing the structure of these ripples.

Our second methodological innovation involves the systematic use of oriented areas (discussed in the Oriented Areas section) *as functions of time*. By design, the oriented areas, or other iterated integrals, are *numeric* quantifiers. Each of them associates a single number to a (multidimensional) time series, defined over a time interval. However, one can also consider these time intervals as expanding in time by making their upper bound variable and evaluate the oriented areas on subintervals [*t*_*s*_, *t*], *t*_*s*_ < *t* ≤ *t*_*f*_ of the total observational time interval [*t*_*s*_, *t*_*f*_]. This turns the oriented areas into the functions of *t*. A careful investigation of how the oriented areas evolve can, as it turned out, provide significant insights into the nature of the process. In what follows, we apply the CA computational pipe without any modifications to the time series extracted from the regions of interest (ROIs).

## ANALYTIC APPROACH

The computational procedure of CA, which underlies our findings, exploits pairwise temporal relations between the time series corresponding to different ROIs. These temporal relations are quantified through the so-called *iterated path integrals* as the baseline quantifiers of our observed time series (Chen, 1958). The applicability of the CA pipeline to the analysis of fMRI traces was demonstrated in various studies (Baryshnikov & Schlafly, 2016; Shahsavarani, Abraham, Zimmerman, Baryshnikov, & Husain, 2020; Zimmerman et al., 2018).

Our approach relies on the iterated integrals of the lowest degree, where the outputs are not reducible to the functions of the increments of the time series, that is, on the iterated integrals of order two. These integrals, essentially, represent the *oriented areas* encompassed by the two-dimensional (2D) projections of the trajectory. One might also consider iterated integrals of higher orders. While they tend to become increasingly corrupted by noise, the idea of using iterated integrals of all orders for data analysis is advanced by T. Lyons and his school (Bonnier, Kidger, Arribas, Salvi, & Lyons, 2019).

### CA and Traveling Waves

Computationally, CA is a straightforward and efficient pipeline (we provide greater details on the algorithmic part of CA in the Section 1 of the Supporting Information). One begins with computing the oriented areas:

$$a_{kl}=\frac{1}{2}\int_{t_{s}}^{t_{f}}x_{k}{dx}_{l}-x_{l}{dx}_{k}=\frac{1}{2}\int_{t_{s}}^{t_{f}}x_{k}\left(t\right){\dot{x}}_{l}\left(t\right)-x_{l}\left(t\right){\dot{x}}_{k}\left(t\right)\;dt$$ (1)

encircled by the parametric plots of the time series (appropriately interpolated, − linearly in this study) for any pair of the time series *x*_*k*_(*t*), *x*_*l*_(*t*), where *k*, *l* = 1, 2, …, *n* and *n* is the total number of ROIs. (We remark that Equation 1 is equivalent to Equation 2 below, or Equation 4 in Zimmerman et al. (2018), written in a matrix form. We use here the component-wise formulation to make the presentation more transparent.)

As we have shown previously in Zimmerman et al. (2018), a *positive* oriented area *a*_*kl*_ > 0 is heuristically indicative of *x*_*k*_ being the leading indicator to the follower *x*_*l*_, and conversely, the negative *a*_*kl*_ < 0 indicates that *x*_*l*_ is the leader and *x*_*k*_ is the follower. Note that the oriented areas generated by perfectly correlated or anticorrelated signals vanish, as well as the area spanned by signals with nonoverlapping supports.

To address pairwise leader-follower relationships synergistically, these oriented areas are arranged into the *n* × *n* matrix *A* = (*a*_*kl*_) for *k*, *l* = 1, …, *n* (which is therefore skew-symmetric). This matrix is equivalently provided by the equation:

$$A=\frac{1}{2}\int_{t_{s}<u_{1}<u_{2}<t_{f}}dX\left(u_{1}\right)⊗dX\left(u_{2}\right)-dX\left(u_{2}\right)⊗dX\left(u_{1}\right)$$ (2)

where the trajectories *x*_*k*_(*t*) have been collected together into a vector *X*(*t*). This matrix *A* is referred to as the *lead matrix*, and its spectral decomposition allows one to assess the *collective* temporal interdependencies between the components ${\left\{x_{k}\right\}}_{k=1}^{n}$ (a precise mathematical definition is available under the Lead Matrices section along with plots of a representative example).

The nonzero eigenvalues of the lead matrix consist of the purely imaginary pairs ±*iλ*_*s*_—for some, real *λ*_*s*_—which we assume are ordered by size: ∣*λ*_1_∣ ≥ ∣*λ*_2_∣ ≥ …. The eigenvectors corresponding to the conjugate eigenvalues ±*iλ*_*s*_ are complex conjugate (and necessarily have nonvanishing imaginary components).

How fast the absolute values of the spectrum of the lead matrix decrease is a heuristic indicator of the dominance of the temporal interaction network by a small number of dynamic patterns. Observing just a few pairs of large (in absolute value) eigenvalues ±*iλ*_*s*_ indicates a good approximation of the observations by just a few propagating ripples.

### Ripples

In some classes of cyclic (not necessarily periodic) signals—defined and referred to as the Chain of Offsets Model (COOM) below—*the components of the eigenvector corresponding to the eigenvalues with the largest (in absolute value) eigenvalue ∣λ_k_∣ line up along an ellipse in the complex plane in cyclic order corresponding to the one according to which the time series follow each other in the underlying system* (we provide further details in the Chain of Offsets Model section).

Thus, we pick the eigenvalue with the largest absolute value ∣*λ*_*k*_∣ and select one of the corresponding eigenvectors. We will be referring to such an eigenvector as *the leading eigenvector*. The collection of the components of the eigenvector (these components are complex numbers that we consider as a collection of indexed points in the complex plane) is referred to as *the constellation*. Each of the points in the constellation corresponds to one of the ROIs in our sample. Note that there is some ambiguity as those eigenvectors come in complex conjugate pairs, but in practice, this does not complicate matters, as the signs of the entries of the lead matrix allow one to easily distinguish between two possible orientations.

Under the COOM assumption, we interpret the angular order of the points in the constellation as indicative of the relative order of the nodes—or ROIs—in the wake of the ripple sweeping through the network. (In Section 1 of the Supporting Information, we show, using simulated wave propagation, that in an excitable network with lags, this heuristic remains valid.) It should be noted that, in general, not all nodes would be receiving a particular signal, and for those nodes, the corresponding components of the leading eigenvector will cluster near the origin. This implies that meaningful analysis should first single out the largest parts of the constellation (as defined by an *intrinsic* metric) and focus on those. In the following sections, we show that this leads to a metric defined by quadratic forms.

## RESULTS

### Dominant ROIs

As described in detail in the Materials and Methods section, we generated time series describing the BOLD activities in the 68 ROIs (i.e., from 34 bilateral pairs). These time series were obtained by averaging time series over voxels within those ROIs (defined in Table 1) in each resting-state fMRI (rs-fMRI) recording with a duration of *t*_*f*_ = 864 s. The time series were then centered (setting their time averages to 0) and normalized by their quadratic variation, that is, the sum of squared differences. Oriented areas *a*_*kl*_(·) were then computed using a discretized version of Equation 1 (see comments in the Discretization section).

**Table 1.** Table of 68 ROIs involved in the analysis, showing numbers for leftside regions; rightside regions run their indices as 35 through 68

ROI #	ROI full name	ROI #	ROI full name
1	Banks of superior temporal S.	18	Pars orbitalis C.
2	Caudal anterior cingulate C.	19	Pars triangularis C.
3	Caudal middle frontal C.	20	Pericalcarine C.
4	Cuneus	21	Postcentral C.
5	Entorhinal C.	22	Posterior cingulate C.
6	Fusiform C.	23	Precentral C.
7	Inferior parietal C.	24	Precuneus
8	Inferior temporal C.	25	Rostral anterior cingulate C.
9	Isthmus cingulate C.	26	Rostral middle frontal C.
10	Lateral occipital C.	27	Superior frontal C.
11	Lateral orbitofrontal C.	28	Superior parietal C.
12	Lingual C.	29	Superior temporal C.
13	Medial orbitofrontal C.	30	Supramarginal C.
14	Middle temporal C.	31	Frontal P.
15	Parahippocampal C.	32	Temporal P.
16	Paracentral C.	33	Traverse temporal C.
17	Pars opercularis C.	34	Insula

Here, C, cortex; S, sulcus; and P, pole.

For the full lead matrix *A*, the spectral decomposition was determined and the eigenvector v_*_ corresponding to the largest in absolute value eigenvalue *iλ* was chosen. There may be an ambiguity as to which of the two complex conjugate eigenvectors corresponding to the opposite eigenvalues ±*iλ* to chose. However, selecting a leader-follower pair of components *x*_*k*_, *x*_*l*_ with a well-defined phase shift, and ensuring that the orientation of the pair of complex numbers *v*_*k*_, *v*_*l*_—the *k*-th and *l*-th components of the eigenvector *v*—is positive, resolves it.

**Figure 1.**
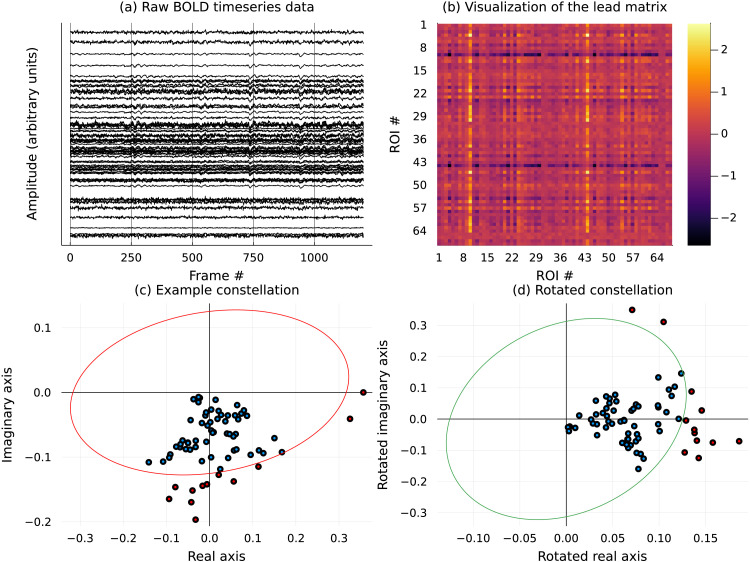
This figure shows an **example** of the preliminary steps in the analysis of resting-state data. (A) The raw BOLD time series is appropriately normalized to create (B) the lead matrix, which captures pairwise leader-follower relationships. ROI numbers are related to names as listed in Table 1. The leading eigenvector of the matrix is entry-wise complex valued and is visualized as a (C) constellation on the Argand plane, which with an appropriate angular normalization yields (D) an imputed order between the ROIs deemed dominant (marked in red) with respect to the leading eigenvector (the order among the red points proceeds counterclockwise starting from the negative *x*-axis in (D)). The concentration of the components of the leading eigenvector in the right half of the complex plane is indicative of the traveling wave patterns, where the system comes to rest between ripples. In panels (C) and (D), we visualize in red and green the best fitting ellipse through the origin (least squares fit).

The components of this eigenvector are complex numbers (indexed by the 68 ROIs). The scatterplot of these complex numbers are referred to as *constellation*; examples of them are shown in Figure 1.

In the case of a perfectly harmonic time series, such a constellation would necessarily align with an ellipse (referred to as the *constellation ellipse*, CE), except for the components corresponding to the nodes not activated during the passage of the excitation through the network. Those components would collapse to the origin (Baryshnikov & Schlafly, 2016). For a noisy signal, it is beneficial to separate the activated nodes from irrelevant ones by selecting the components farthest away from the origin. It should be emphasized, however, that the distance should be measured with respect to the quadratic form defining the CE, not the standard Euclidean metric.

In this study, we identified the CE or, rather, the positive-definite quadratic form

$$q\left(z\right)={ax}^{2}+2bxy+{cy}^{2},\;z=x+iy$$

on the complex plane (for which the CE is the unit level set {*q*(*z*) = 1}) using least square regression. The resulting norm ∣*z*∣_*q*_ ≔ *q*(*z*)^1/2^ allows us to order the ROIs (points in the constellation) according to their distance defined in terms of *q*: The point of the constellation with the largest norm has rank 1, and so on—see Figure 1 for a representative example.

Naturally, these quadratic norms ∣·∣_*q*_ differ from recording to recording. One can, however, construct an *average ranking* over all recordings, derived from the constellations associated with the leading eigenvalue of the lead matrices. The points of the *low* average rank (i.e., of large ∣·∣_*q*_ distance to the origin) are expected to be exposed to the presumed excitation rippling through the cortex, while those of *high* average rank (i.e., closer in ∣·∣_*q*_ distance to the origin) are heuristically not affected by the ripple.

**Figure 2.**
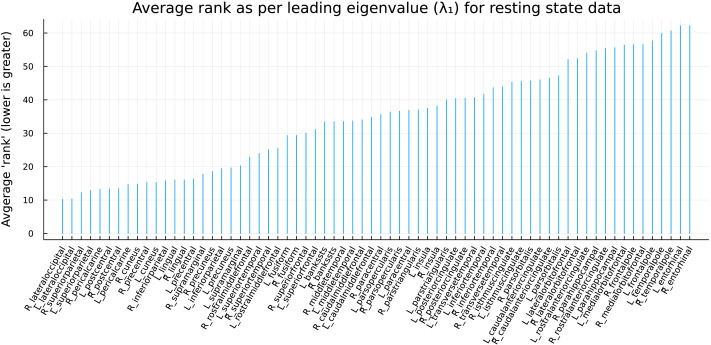
The ranking of the ROIs in terms of their elliptic distance for the first eigenvalue. The elliptic distance is a measure of how far an ROI is from the origin in its constellation (i.e., how dominating the signal is within the collection with respect to the first eigenvalue). The ranking depicted here is obtained by considering constellations from all resting-state scans across all subjects and, thus, is the *average rank*.

Again, these ranks are computed in terms of the constellations corresponding to the *leading* eigenvalues in each recording; one can also consider the ranks corresponding to the second highest eigenvalue and so on, recovering potentially independent ripples. Figure 2 summarizes the average rankings for each ROI corresponding to the first eigenvalue (the results for the second eigenvalue is provided in the Supporting Information).

From hereon, we focus primarily on the leading eigenvalues (with the largest absolute value, i.e., *λ*_1,2_ in our convention), but one can extend the same procedures to the next largest in magnitude eigenvalue as well.

### Ordering the ROIs Phases

Using the average ranking chart in Figure 2, we can choose a cutoff, akin to choosing a knee point from an eigenvalue plot in principal component analysis, to limit the number of ROIs affected by the leading (i.e., corresponding to the leading eigenvalue) ripple for further analysis.

Once a subset of ROIs is chosen in this way, one can estimate the **ordering** of those *significant* ROIs by examining the arguments of the complex valued entries in the eigenvector corresponding to them. As explicated in the Ripples section, if the nodes are excited by similar waveforms delayed by a collection of the phase shifts, CA is able to extract those phase shifts, and, correspondingly, an approximate *order* in which the ROIs are excited as the ripple passes through the network (Baryshnikov & Schlafly, 2016; Zimmerman et al., 2018).

This phase shift extraction is especially intuitive when the constellation is clustered in a part of the complex plane, as on the bottom left display of Figure 1. In this situation, one can reposition the axis corresponding to the zero phase so that it points away from the center of mass of the constellation. In this way, the ordering of the shifts coincides with the ordering of arguments of the (complex) components corresponding to the ROIs, normalized to vary from −*π* to *π*. This is shown on the bottom right display in Figure 1, which is obtained from the bottom left display by the rotation aligning the center of mass of the constellation with the positive real ray.

Therefore, for each eigenvalue, and each threshold separating the ROIs with high excitation levels with respect to this eigenvector, one derives the order in which the ripple captured by that eigenvalue excited the nodes.

**Figure 3.**
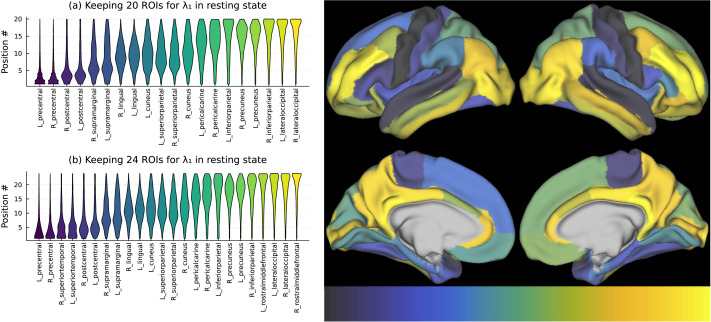
(A, B) This figure shows the statistics of the imputed ordering for 20 and 24 highest ranked (in terms of elliptic rank) ROIs. The right panel shows a visualization created using the freely available Connectome Workbench visualization software (Marcus et al., 2011) of the propagation of an hypothesized wave across cortical regions by shading them according to their positions in the imputed cyclic ordering (keeping all 68 ROIs).

**Figure 4.**
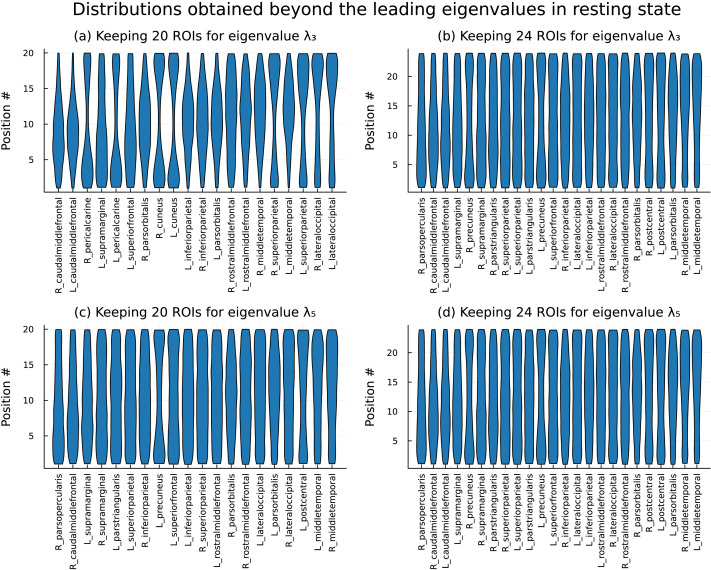
(A–D) This figure shows the distribution of the imputed orderings when keeping the top 20 or 24 ranked ROIs indicated by the *second* and *third* leading eigenvalues *λ*_3_ and *λ*_5_. One clearly see that the statistics imply that the ordering becomes much less reliable and more fluid as we consider lower in magnitude eigenvalues for analysis.

Again, these obtained orderings change across recordings, being impacted by the subjects’ and experimental conditions’ variability and noise. However, the statistical distributions of those phase shifts are indicative of a well-defined ripple, propagating through the cortex. To this end, we derived the histograms of the frequencies with which any given ROI appears in a particular position in the ordering of the phase shifts derived from the constellation. Those histograms, depicted as the violin plots in Figure 3, are shown for the ranking thresholds of *k* = 20 on the top left display of Figure 4. The ordering of the ROIs is by the *average* positions in the order of phase shifts.

It is immediate that these frequencies are indicative of a ripple passing through the selected significant ROIs. Among remarkable features of this plot is the *symmetry*: The bilateral pairs of ROIs are excited at the same stages of the ripple’s passage. Anatomically, the direction of the imputed ripple is shown on the top right display of Figure 3.

Changing the cutoff thresholds does not qualitatively change the observed pattern. The lower part of Figure 3 shows the result for the cutoff at *k* = 24 ROIs. Again, the excitation is symmetric, and the patterns of propagation are anatomically similar to what was observed for *k* = 20 ROIs.

If one attempts to repeat the analysis of the excitations’ passage using the higher eigenvalues (i.e., ±*iλ*_*s*_, *s* ≥ 2), one encounters loss of directionality. The violin plots become disordered and lacked any discernible structure; see Figure 4 with the violin plots for the second and third largest eigenvalues.

### Pairwise Time Series Analysis

Returning to the pairwise temporal interrelationships between ROIs *k* and *l*, we examined the time evolution of their leader-follower dynamics as captured by the oriented areas treated as functions of time,

$$a_{kl}\left(t\right)=\frac{1}{2}\int_{0}^{t}x_{k}{dx}_{l}-x_{l}{dx}_{k}.$$

The overall increment of *a*_*kl*_(*t*) over the interval of observation is the *a*_*kl*_ entry of the lead matrix *A*, but how this increment is reached supplies us with additional information. While the overall trend or increment is a succinct indicator of the average leader-follower relationship, the dynamics of the oriented area signal can provide insights into the dynamics of the interaction between the corresponding brain regions.

We noted primarily three kinds of dynamics that oriented area signals exhibited from the dataset.

1. Neutral dynamics with low-level fluctuations near zero.
2. Steady, nearly monotonic overall increment or decrement.
3. Steady increment or decrement punctuated by *role reversals* in the leader-follower relationship.

**Figure 5.**
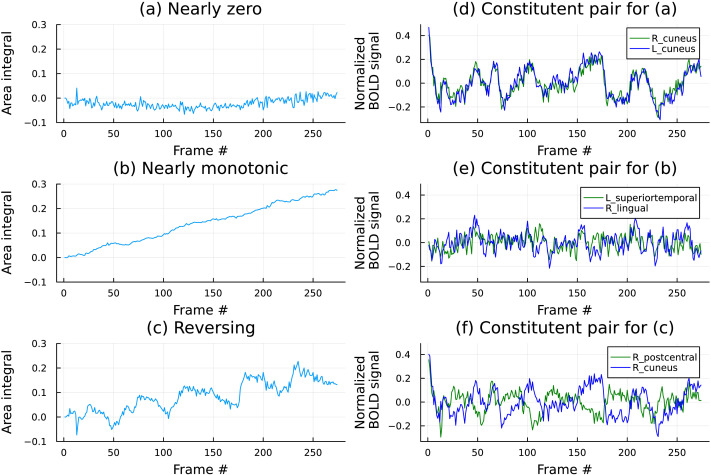
The figures above show three major types of dynamics that the oriented area signals between pairs of regions exhibited. (A, D) The panel at the top left shows the oriented area obtained from L_cuneus and R_cuneus (depicted on the top right). This is an example of a bilateral pair of brain regions exhibiting the first kind of behavior in the list above. (B, E) The second panel shows that ROIs labeled L_superiortemporal and R_lingual maintained the same pairwise leader-follower behavior throughout the scan, typical of the second behavior listed above. (C, F) Finally, the bottom panel shows a pair exhibiting an overall increment over the scan duration punctuated by reversals, that is, the change in the behavior of the area integral from increasing to decreasing and vice versa between ROIs labeled R_postcentral and R_cuneus ROIs.

The three kinds of dynamics are illustrated in Figure 5 using examples from the data.

One would expect the first kind of dynamics for pairs of time series that are strongly correlated (or anticorrelated), and not unexpectedly, we observed them between the pairs of bilateral ROIs. The steady dynamics of the second type manifested itself frequently in the recordings of the resting-state sessions. More interestingly, the third intermittent kind of dynamics was observed in a large fraction of the *task* datasets. One can note, by observation, the timings of the reversals of oriented area accumulations, and it becomes readily apparent that they appear in a regular fashion and are quite localized.

(We should remark that the behavior exhibited on the bottom row of Figure 5 is unlikely to be reproduced by a low-complexity autonomous 2D system and is highly indicative of external excitations, causing the reversals of the oriented areas.)

The next two subsections deal with the detailed analysis of these observations, where we devise some tools to quantify these phenomena.

### Reversals in the Task Paradigm

**Figure 6.**
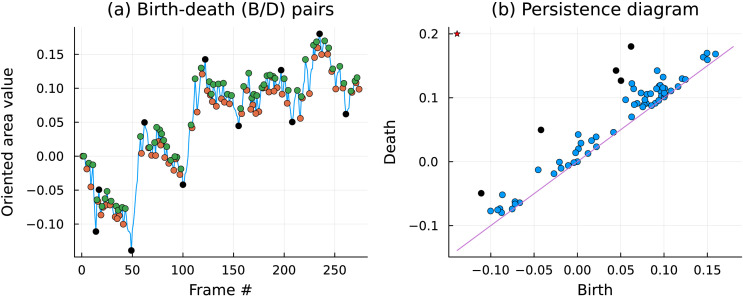
Example of how tools from persistent homology is used for analysis: (A) All local minima and maxima of a time series are marked to create a birth-death diagram, which is, in turn, used to create a (B) *persistence diagram*. The points farthest from the diagonal are used to inform the selection of the longest lived features (black points in the left panel). The global minimum is paired with a maximum at infinity (starred marker). Together, the two panels show the selection of six pairs in the above figure.

To further examine the phenomenon of dynamic fluctuations observed in the oriented area signals, we accumulated over all pairs of ROIs the time stamps at which a change in the direction of the oriented area occurred. While, in general, this happens at the (local) maxima and minima, one can filter out the small-scale jitter using the toolbox of *Persistent Homology*, briefly outlined in the Section 2 of the Supporting Information. One does this by coupling the local minima and maxima in a well-defined way. The heights *b*, *d* of such coupled minimum and maximum are thus referred to as the *birth* and *death* of a feature and are visualized as points on the (*b*, *d*) plane, as shown in Figure 6. We interpret the features with the largest span (i.e., the points furthest from the diagonal {*b* = *d*}) as indicative of the reversals. The coupled pairs whose span *d* − *b* is high are said to be *persistent* features in the data (see Ghrist, 2008 for more details).

**Figure 7.**
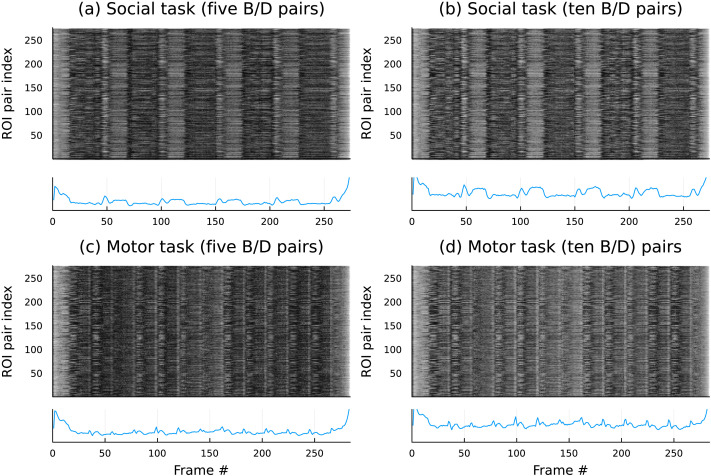
Changes in oriented area dynamics as observed from the two task paradigm datasets analyzed. (A, B) The upper panels show the social task results, (C, D) while the lower ones correspond to a motor task. Each of the columns are obtained by choosing to keep *p* = 5 or *p* = 10 longest lived bars (see Figure 6) in either the motor or the social task. Finally, the line plots show the number of ROI pairs that recorded a birth or death (on the *y*-axis), while the *x* coordinate of all plots indicate the axis of time (in units of fMRI frames).

We mark the time stamps of the *minima* of these persistent features. Combining all time stamps of such persistent features across all oriented area time series (corresponding to pairs of ROIs), we visualize them using a carpet plot as shown in Figure 7 (for task paradigm data).

One prominent feature of the social task plots is the regular patterns occurring approximately every 52 frames (i.e., every 0.72 s × 52 = 37.4 s). The documentation (Barch et al., 2013) revealed that the intervals between social task segments were 38 s. A similar analysis of the motor task data showed approximately 11–13 repeating epochs within the duration of the observation. Once again, the documentation recorded that for motor tasks, the participants were presented with stimulus in 13 blocks (10 movements, each were 12 s long, and with three 15-s fixation blocks). The strongest indications of a reversal consistently appeared at the onset and offset of the task blocks, which may reflect a switching between task-positive and task-negative systems at these transitions between task and rest.

**Figure 8.**
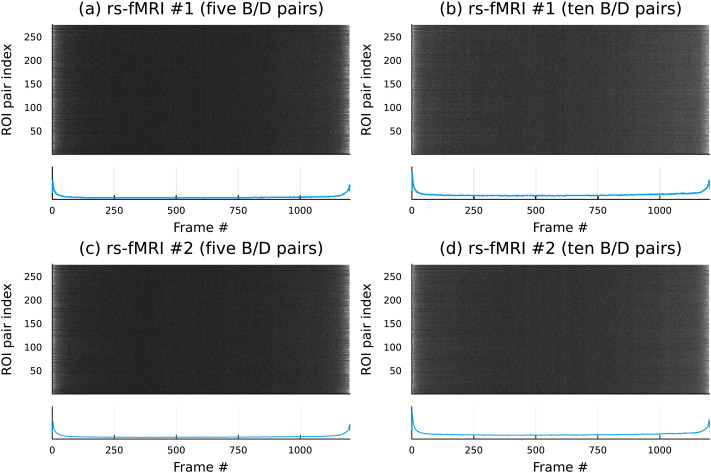
In marked contrast with Figure 7, the rs-fMRI scans show very little dynamic changes, implying that the largest bars for the oriented area plots for resting-state sessions are spread out over the duration of the session without any notable patterns. (A–D) The dataset consisted of two resting-state scans per participant, which are labeled as rs-fMRI #1 and rs-fMRI #2.

A similar analysis of the resting-state sessions revealed that pairwise activities, as indicated by the above outlined analysis, were, indeed *at rest*; see Figure 8.

### Burstiness in Time Series

**Figure 9.**
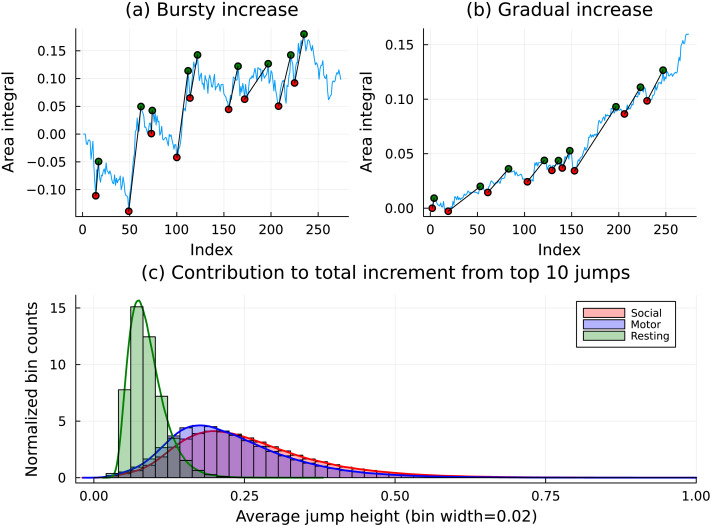
This panel captures the differences between three different types of MRI scans in the data (resting state and motor or social tasks). As mentioned in the Pairwise Time Series Analysis section, typical pairs of brain regions exhibited three different behaviors in their leader-follower activity. (A, B) The top two panels show pairs that exhibit the same level of overall oriented area increments but achieve it in markedly different manners. (C) The bottom panel shows the distribution of the contributions made by the top 10 persistent features to the overall jump. We see that the task paradigm data was far more *bursty* in nature than the resting-state data.

To analyze *burstiness* in the time series, we extended the concepts outlined above to arrive at a mechanism for quantifying the difference between the resting and task paradigms, as evidenced by the activity of pairs of brain ROIs. One salient observation that arose from the study of oriented areas as functions of time was that activity between some regions tended to be much more consistent than others. These are exhibited by a gradual increase in the overall oriented area integral. The less-consistent pairs, on the other hand, contributed to the total increment of the oriented area in jagged “bursts.” Figure 9 illustrates this phenomenon in its top two panels. The top left image shows a pairwise oriented area integral dominated by the jumps from the deep local minima. The top right image, however, shows another pair of brain regions, where the total accumulated oriented area is similar to that of the left display, but does that accumulation in a more *gradual* fashion.

We have marked on either plot the 10 longest persistent features observed in the time series calculated according to the technique described in the previous section. Using this heuristic, we define *the burstiness* of the oriented area function as the total sum of the lengths of the 10 longest bars in the persistence diagram. (We remark that the results are robust with respect to the number of the longest bars chosen for analysis: Selecting 8 or 20 longest bars leads to essentially identical outputs.)

Remarkably, the distributions of the *burstiness*, computed for all pairs of ROIs and scans, are dramatically different for the resting-state and the tasked sessions. As one can see in the bottom panel of Figure 9, the burstiness for the resting state is far lower than that of either motor or social tasked sessions. On the other hand, the distributions of burstiness for the motor and social tasks are very close. Note that all time series were energy-normalized, so that the low burstiness of the resting state cannot be attributed to different scales of the cortical activity.

## DISCUSSION

We believe that CA stands as a promising avenue for gaining novel insights into the dynamics of FC, thereby enhancing our understanding of brain networks and their interactions. Our research findings substantiate this perspective by revealing a distinctive temporal ordering in the resting-state activity of brain regions, particularly along a spatial axis suggestive of hierarchical cortical organization. The results provide empirical support for the existence of cortical waves propagating between transmodal areas and the primary visual cortex, highlighting the relevance of CA in uncovering these patterns. Additionally, the identification of short periods of sustained lead-lag relationships in oriented areas further emphasizes the time-dependent flow of information underlying the dynamic aspects of hemodynamic measures. This temporal organization that is characterized by abrupt “bursts” rather than gradual changes underscores the dynamics of the brain’s functional architecture in the task paradigm. Importantly, we observed noteworthy shifts in the direction of wave propagation during specific tasks, presenting repeated reversals that contrast with the observed steady patterns in the resting state.

There are several other well-regarded computational techniques aimed at the discovery of the temporal interrelationships between brain regions. The *lagged correlations* method of Mitra, Snyder, Blazey, and Raichle (2015) infers, as its first step, the relative lags between pairs of time series, defined as the time shift that maximizes the correlation coefficient between them. (The resulting skew-symmetric matrix can be considered a nonreparameterization-invariant version of our lead matrix.) Next, they adjust this matrix by removing the mean lag for each time series. Then, they extract the leading singular vector so as to determine the sequence of lags best compatible with their modified lag matrix.

The approach of extracting the leading singular vector from a delay matrix is used broadly, with differently defined delay matrices. For a different example, see Gu et al. (2021), where the time series are sliced into segments by the local minima of the overall brain activity, and then within each such segment, the delays are detected, again by analyzing the local peaks of activity for each ROI.

In all these methods, the inferred *time lags* are the key element used in the subsequent numerical procedures. As such, they are inherently dependent on the consistent rhythm of the observed processes. Lagged correlation methods rely on interpolation and windowing to capture the dynamics of FC, which are sensitive to time delay estimation methods, autocorrelation (Shahsavarani et al., 2020), sampling variability (Raut et al., 2020), and parameters such as window length and window shift (Shakil, Lee, & Keilholz, 2016). In contrast, CA offers an approach with finer granularity resolution, and without the aforementioned sensitivities or assumptions regarding stationarity, state duration, or state transition, all following the principled reliance on *reparametrization-invariant* tools.

Further, the spectral analysis under CA is critically different from that of the extant methods. To extract the temporal ordering of the signals, we rely on the inherently complex valued eigenvalues of the skew-symmetric lead matrix (see the Cyclicity Analysis section), while other methods preprocess it to extract its singular vectors, which are then interpreted as the delay vector. From these perspectives, the CA is a method of extraction of the temporal ordering of the observed signals. which is radically different from the existing ones.

Although cortical waves have been noted at much higher frequencies, detecting them in fMRI data at subhertz frequencies with CA tools is a novel observation. At higher frequencies, cortical waves of oscillatory activity are thought to contribute to a spatiotemporal framework for neuronal communication by coordinating a range of synchronizing patterns (Das et al., 2022; Muller et al., 2016, 2018; Roberts et al., 2019; Rubino, Robbins, & Hatsopoulos, 2006; Townsend et al., 2015; Zhang et al., 2018). Although spike trains of individual neurons have shown to be coupled to both slow and fast time scales (Okun, Steinmetz, Lak, Dervinis, & Harris, 2019), the temporal duration of these more typical cortical waves tends to be on the order of tens to hundreds of milliseconds rather than the subhertz frequencies, which BOLD signals are able to resolve. Nevertheless, over the past decade, a growing body of studies has observed BOLD signal flow across cortices (Gu et al., 2021; Hindriks, Mantini, Gravel, & Deco, 2019; Ma & Zhang, 2018; Majeed et al., 2011; Mitra, Snyder, & Raichle, 2020; Raut et al., 2021). Taken together, these studies provide evidence that there is likely a propagation of the BOLD signal throughout the brain that dynamically carries or reflects longer periods of stable information flow between brain regions. The method that we have outlined here should be a useful tool for investigating these dynamics due to its invariance to the arbitrary nonlinear reparameterizations of the timeline, a feature lacking in the correlation-based methods.

One striking pattern revealed by our analysis is the predominant role of distinct, short-term “bursts” in lead-lag relationships between pairs of brain regions, as opposed to prolonged, consistent stretches of moderate associations. This should be compared with the recent paper by Esfahlani et al. (2020), where the authors investigated the contributions of moment-to-moment BOLD activity to the overall pattern of FC. Similar to our observation, they saw that only a small fraction of frames in the time series explained a significant amount of the variance in the network connectivity. These fluctuations corresponded with activity patterns aligned with fluctuations in the level of default mode and attentional control network activity, which are often viewed as in opposition to each other (Anderson, Ferguson, Lopez-Larson, & Yurgelun-Todd, 2011).

This opposition between the default mode and attention networks has a strong overlap with the idea of principal gradients of macroscopic cortical organization in the brain (Margulies et al., 2016; Samara, Eilbott, Marguilies, Xu, & Vanderwal, 2023). According to this framework, a topography of connectivity patterns is reflected in the spatial distances between the so-called “higher” areas of the brain, where the more multimodal, abstract, predictive information is encoded, and “lower” areas, such as the primary sensory/motor regions. This primary connectivity gradient predicts the positions of canonical resting-state networks, which are viewed in this framework as reflecting representational hierarchies rather than distinct modules. In other words, resting-state networks are reflective of the temporal phase of propagating patterns. The functions associated with various cortical networks were correlated with the level of the hierarchy of sensory or motor processing. Also, Raut et al. (2021) observed slowly propagating waves of activity measured with fMRI in humans, which were associated with cyclic fluctuations in arousal. They then replicated the result in macaque monkeys using hemisphere-wide ECoG. Critically, they found that functional networks maintained phase shifts relative to one another in their relation to autonomic arousal, rather than specific time delays. This result validates the usefulness of a time reparametrization-invariant analytical method like the one we present here.

The results of CA, as applied to the HCP dataset, revealed some unexpected findings, which should be investigated in future studies.

In the context of the resting-state scans, we see ordering in the direction moving along the cortical hierarchy, through association areas toward the visual regions (see Figure 3). The precentral gyrus activity leads, and the lateral occipital cortex follows. Compared with the intermittent bursts in the task state, wave here follows a reverse hierarchy, moving from association areas toward the visual processing regions. Interestingly, our results echo those by Gu et al. (2021), which found that while the global propagation pattern follows the cortical hierarchy at the network level, there may be a reverse hierarchy within the sensorimotor networks (e.g., ending at the lateral occipital cortex rather than the pericalcarine cortex). The temporal ordering between these regions is steady and nearly monotonic. It is possible that during resting state, brain activity is mostly internally generated as in mind-wandering. Possibly because there is no stimulus to attend to, activity mostly occurs in the higher-to-lower direction in the hierarchy, reflecting endogenous processing.

In the context of the social task (see Figure 7), we observed a strikingly different pattern. This task consisted of five blocks, each consisting of a period of watching a video and then judging whether the objects in the video appear to perceive the other objects’ feelings and thoughts. In this task, we still observed strong directional constraints, but which brain area is the leader and which is the follower is shifting over time. This may be interpreted as periods of time that switch between internally generated (endogenous) activity and externally generated (exogenous) activity, which may correspond with periods of watching the videos and periods of making judgments of the objects’ intentions within the video. The direction of the leader-follower relationships is also much burstier compared with the direction during the resting-state data.

The pattern of activity related to the motor task was also bursty and more similar to the social task than the resting-state task (see Figure 7). Here, the participants were presented with 3-s cues to perform 12 motions. There are 10 motor blocks and three 15-s fixation blocks in each run. Like in the social task, we observed switches between the leaders and the followers during the run. However, it is more difficult to match the activity observed to events in the task. This may be because the cuing events (3-s long) and motor movement events (12-s long) are too short to be adequately captured by the low-frequency movement across the brain. We may be observing some aliasing in the directionality of the lead-lag signal. It is also possible that in the motor task, there is more separability between activities belonging to hierarchical visual processing and motor processing.

Our analysis identified a temporal ordering that primarily propagated in opposite directions along an axis resembling the principal gradient of the brain’s spontaneous activity identified by Margulies et al. (2016). However, it should be noted that our analysis focused on analyzing the first eigenvector of the lead matrix, and this favors patterns of activity from subjects having high ∣*λ*_1_/*λ*_3_∣ ratios in the lead matrices. Thus, our choice likely selected for subjects with strong leader-follower activity along a single direction. It is possible that the analysis of the other eigenvectors would reveal a pattern of temporal ordering moving in other directions, reflecting other kinds of neural dynamics, such as the signaling across hemispheres.

In this paper, we deployed CA to detect transient processes in the brain. This new approach complements recent advancements in effective or directional connectivity research. The method outlined here has intrinsic advantages due to its lack of assumptions regarding the temporal properties of the BOLD signal dynamics. It does not assume the stationarity of the time series or specific properties of latency, state duration, or state transitions, which could bias correlational, spectral, or lag-based approaches. We have shown in group data a primary propagating wave of BOLD activity during resting state along a spatial axis related to cortical hierarchical organization. We further observed that these propagating waves appear to switch directions in a task-dependent manner.

The findings reported here open a wide range of future research directions. Future work should apply CA techniques to other measures of human brain activity that are more reflective of direct neural activity, including EEG, ECoG, the fast optical signal, and magnetoencephalography. Applying CA to multiple sensing modalities may help clarify the relationship between patterns observed in the fast temporal domain of neuronal activation and the longer duration patterns observed in the BOLD signal, which may be more reflective of broader stable states of the whole-brain function.

## MATERIALS AND METHODS

From the Human Connectome Project 1200 Release (Glasser et al., 2013; Van Essen et al., 2012), we considered 889 denoised minimally preprocessed participants who completed all the structural, resting-state, and task fMRI sessions using a customized Siemens Skyra 3 T scanner. Of those, 27 were excluded from the analysis for the segmentation issues noted in the HCP Quality Control process or functional preprocessing errors reported in the HCP Data Release Updates. After exclusion, 862 participants remained for analysis who ranged in age from 22–45 years and included 464 females. We used the Connectome Workbench software to extract time series from ROI information in the fMRI parcellations available for download.

### Preprocessing

The ROIs extracted were 34 in number (bilateral; therefore, total *N* = 68) and are listed in Table 1. The parcellated output files were further processed in Python/R to create time courses for the regions listed in Table 1. At a time repetition of 720 ms, for the rs-fMRI scans (numbering four per participant), this resulted in a set of arrays *D*, with elements of dimension 68 × 1, 200 that were fed into the CA pipeline. (Here, 1, 200 is the number of fMRI frames; the total session’s duration, therefore, being 14.4 min). Further details of the scan protocol are available online from the HCP Project Consortium. The top left panel in Figure 1 shows a representative of BOLD signals in *D*.

The HCP dataset includes fMRI scans recorded under different task paradigms. Such scans recorded during the progression of certain motor and cognitive tasks were also included in part of our analysis. The two task protocols are as follows—for the **motor task** (Barch et al., 2013):

Participants are presented with visual cues that ask them to tap their left or right fingers, squeeze their left or right toes, or move their tongue to map motor areas. Each block of a movement type lasts 12 s (10 movements), and is preceded by a 3 s cue. In each of the two runs, there are 13 blocks, with 2 of tongue movements, 4 of hand movements (2 right and 2 left), 4 of foot movements (2 right and 2 left) and three 15 s fixation blocks per run.

whereas, for the **social task** (Barch et al., 2013):

Participants are presented with short video clips (20 s) of objects (squares, circles, triangles) either interacting in some way, or moving randomly. These videos were developed by either Castelli and colleagues (Castelli, Happé, Frith, & Frith, 2000) or Martin and colleagues (Wheatley, Milleville, & Martin, 2007). After each video clip, participants chose between 3 possibilities: whether the objects had a social interaction (an interaction that appears as if the shapes are taking into account each others feelings and thoughts), Not Sure, or No interaction (i.e., there is no obvious interaction between the shapes and the movement appears random). Each of the two task runs has 5 video blocks (2 Mental and 3 Random in one run, 3 Mental and 2 Random in the other run) and 5 fixation blocks (15 s each).

### Processing

**Figure 10.**
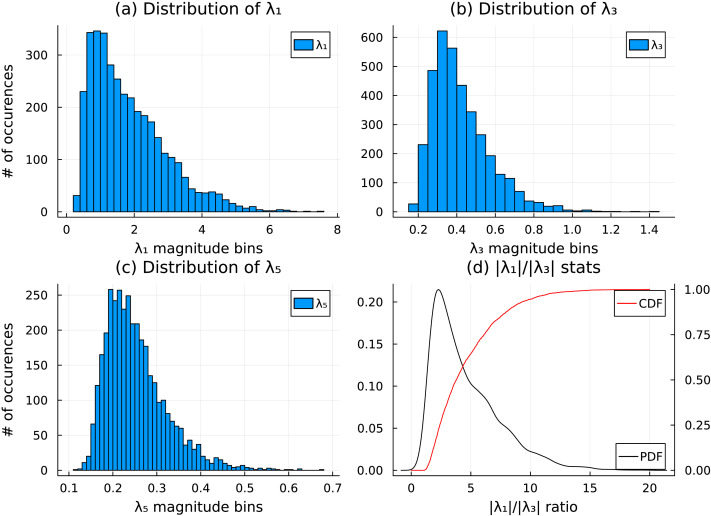
(A–D) Frequency of the absolute values of four largest nonconjugate eigenvalues of the lead matrices in the data (left) along with the cumulative histogram of the observed *λ*_1_/*λ*_3_ values (right). Higher *λ*_1_/*λ*_3_ values indicate that the data closely follows the COOM assumption.

For CA, lead matrices were generated from the time courses and their eigenstructure were analyzed. These matrices have the dimension 68 × 68, with each (*i*, *j*) entry denoting the average leader-follower relationship between ROI *i* and ROI *j*. See the right panel in Figure 1 for a representative example of a generic lead matrix. The ∣*λ*_1_∣/∣*λ*_3_∣ ratio (here, *λ*_*k*_ are eigenvalues of the lead matrix), a measure of the dominance of the rank 2 approximation (described under the Chain of Offsets Model section), was computed for all lead matrices. See Figure 10 for relevant statistics. (Note that *λ*_1_ = $\bar{\lambda }$_2_ since the lead matrix is real and skew-symmetric by construction.)

### Cyclicity Analysis

Recall that the CA pipeline builds on the iterated integrals of *second order* (Zimmerman et al., 2018).

#### Oriented areas.

**Figure 11.**
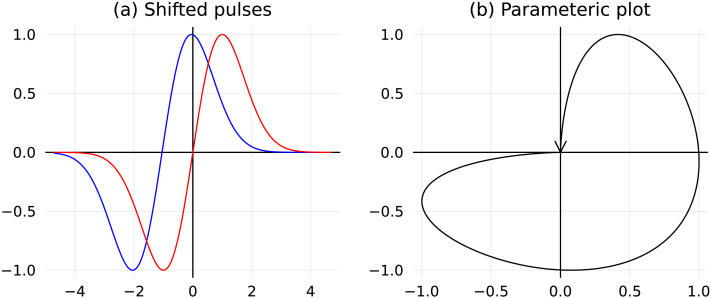
The *oriented* or *algebraic* area encompassed by a pair of time series. (A) The left pane show a pair of time series *x* and *y* that are time-shifted copies of each other (abscissa is time). (B) When plotted *against* each other, the right panel shows that they form a closed contour on the *x* − *y* plane.

Such iterated integrals, not expressed in terms of the iterated integrals of the first order, are known as the *oriented areas*. It relies on an intuitive interpretation of the oriented area encompassed by a curve in the plane, parametrically represented by a pair of trajectories, *x* and *y*. Consider a function of time *x*(*t*) shaped as a pulse (blue curve in Figure 11), as well as its time-shifted copy, *y*(*t*) (red curve). We interpret the interrelationship between *x* and *y* as a *leader-follower* one: *x* leads and *y* follows. Now, to express this relationship in a reparameterization-invariant fashion, we render it using just the parametric plot (taking into account the orientation of the resulting curve in the (*x*, *y*) plane). The key observation now is that **when plotted against each other, these two trajectories enclose a region** *R* **of the plane of positive area** (as shown in Figure 11).

Consider a *closed* parametric curve (*x*(*t*), *y*(*t*)) ∈ ℝ^2^, *t* ∈ [*t*_1_, *t*_2_] with piecewise differentiable functions *x*, *y* as the components. This curve partitions the plane into the open connected domains. Within each of these domains, the winding number of the curve around a point is well-defined: One can imagine an observer located within such a domain, tracing the point of the trajectory; the winding number counts the total number of turns the observer makes (if the curve winds around the point *counterclockwise*, the point’s winding number is positive; if *clockwise*, negative). The sum of the areas of those domains, weighted by their winding numbers, is called the *algebraic or oriented area* encircled by the curve.

Alternatively, the algebraic area can be calculated using the representation

$$\text{area}\left(R\right)=\frac{1}{2}\int_{C}xdy-ydx=\frac{1}{2}\int_{t_{1}}^{t_{2}}x\left(t\right)\dot{y}\left(t\right)-y\left(t\right)\dot{x}\left(t\right)\;dt,$$ (3)

implying that the oriented area is an iterated integral of order 2 (here, *C* is the curve, serving as the contour of integration, oriented by the time). Note that this area is *signed*; in particular, reversing the orientation of the curve or interchanging the variables flips the sign of the oriented area.

Note that the quantity in Equation 3 behaves differently from the correlation coefficients. While the correlation of two signals is largest when they are proportional, the oriented areas are intrinsically antisymmetric, so that it necessarily vanishes on a pair of proportional functions. On the other hand, if the time supports of the two signals do not overlap in time, their oriented area measure is also zero.

#### Discretization.

In practice, the integrals are replaced by the discretized sums requiring an estimate of the resulting error. From the analytical viewpoint, one needs to evaluate the dependence of the integrals (Equation 1) expressing the oriented areas on the variations of the integrands *x*_*k*_, *x*_*l*_: how they change when passing to the functions $x_{k}^{\prime }$, $x_{l}^{\prime }$. One can a priori assume that the error terms *e*_*k*_ ≔ $x_{k}^{\prime }$ − *x*_*k*_, *e*_*l*_ ≔ $x_{l}^{\prime }$ − *x*_*l*_ have bounded *C*^1^ norms *C*_*k*_, *C*_*l*_, respectively, and vanish at the discretization points Δ*n*, *n* = 0, …, *N*.

Under these conditions, an elementary estimate can be performed that leads to

$$|a_{kl}^{\prime }-a_{kl}|\leq 2C_{k}C_{l}\Delta .$$

Thus, the error decreases with Δ, justifying the discretization step.

#### Lead matrices.

In isolation, oriented areas just provide information about the pairwise relationships between two time series. To capture the collective information a multidimensional trajectory represents, we need to aggregate these data in a certain way.

For a trajectory in an *n*-dimensional Euclidean space ℝ^*n*^, we arrange the oriented areas into a square *n* × *n* matrix (where *n* is the number of time series observed). This matrix, whose (*k*, *l*)-th entry is the oriented area spanned by the pair *x*_*k*_, *x*_*l*_ of the time series, is referred to as the *lead matrix* (Baryshnikov & Schlafly, 2016). We remark that this matrix is obviously *skew-symmetric*, implying particularly that its eigenvalues form pairs of purely imaginary numbers, ±*iλ*_*s*_, *λ* ∈ ℝ, *s* = 1, 2, …, *n*, and the corresponding eigenvectors form complex conjugated pairs with necessarily complex valued components.

#### Chain of Offsets Model.

Consider now the situation where the coordinates *x*_*j*_, *j* = 1, …, *n* of the trajectory **x**(**t**) correspond to the same function (which we will interpret here as the function on the internal clock space, a circle) *ϕ* : *S*^1^ → ℝ, just offset by a different phase. In other words,

$$x_{k}\left(t\right)=a_{k}\phi \left(t-\alpha_{k}\right);\;\alpha_{j}\in S^{1},\;k=1,\ldots ,n.$$ (4)

We will be referring to this model as the COOM.

The most basic situations leading to this model are instances of a *periodic traveling wave* passing through a terrain; see, for example, Sherratt and Smith (2008). We note also that traveling waves are routinely used to describe brain dynamics, particularly when measured using MRI, see, for example, Aquino, Schira, Robinson, Drysdale, and Breakspear (2012) and Roberts et al. (2019). Traveling waves are described by the propagation pattern given by the equation

$$U\left(\xi ,t\right)=a\left(\xi \right)u\left(\omega t-h\left(\xi \right)\right),$$ (5)

where *t* is the time variable, *ξ* is the spacial variable, *ω* the system frequency, *a* is the site-dependent amplitude scale, and *h* is a function capturing the spatial directionality of the wave; thus, for the flat wave, *h* is a linear function, while for, say, a spherical wave, *h* is the distance from the excitation center.

If a finite number of sensors is measuring the signal at a collection of points {*ξ*_*k*_} where *k* = 1, …, *n*, with an uncertain scale, then the observed signals will be time series

$$x_{k}=a\left(\xi_{k}\right)u\left(\omega t-h\left(\xi_{k}\right)\right)=a_{k}\phi \left(t-\alpha_{k}\right),$$

where the seed function is given by *ϕ*(*t*) ≔ *u*(*ωt*) and the offsets are given by *α*_*k*_ = *h*(*ξ*_*k*_)/*ω*.

One should remark that the reparameterization-invariant nature of our tools makes COOM applicable when the waves coming through the substrate parameterized by *ξ* need not be necessarily overlapping. The whole formalism works for a sequence of solitary waves, between which the system comes to rest.

#### Lead matrix for COOM.

Under the COOM, the lead matrix can be readily computed in terms of the Fourier coefficients *c*_*m*_, of the seed function *ϕ*: It is given by

$$A_{k,l}^{\phi }=2\pi a_{k}a_{l}\sum_{m\geq 1}m{|c_{m}|}^{2}\sin\left(m\left(\alpha_{k}-\alpha_{l}\right)\right)$$ (6)

In particular, the lead matrix given by Equation 6 decomposes into the sum of *rank 2* skew-symmetric matrices with entries

$$A_{k,l}^{\left(m\right)}=m{|c_{m}|}^{2}a_{k}a_{l}\sin\left(m\left(\alpha_{k}-\alpha_{l}\right)\right)$$

If one of the coefficients in the Fourier series for *ϕ* dominates, the skew-symmetric matrix *A*^*ϕ*^ is well approximated (in Frobenius norm) by the rank 2 matrix *A*^(*m*)^, with each (*k*, *l*) entry given by:

$${\left({|c_{m}|}^{2}a_{k}a_{l}\sin\left(\alpha_{k}-\alpha_{l}\right)\right)}_{kl}={|c_{m}|}^{2}{\left(v_{k}u_{l}-v_{l}u_{k}\right)}_{kl},$$

where *u*_*k*_ = *a*_*k*_ cos(*α*_*k*_), *v*_*k*_ = *a*_*k*_ sin(*α*_*k*_). In general, if a skew-symmetric operator of rank 2 is represented as *Q* = *u* ⊗ *v* − *v* ⊗ *u* (where ⊗ denotes the outer product of vectors), then one can see that *w* = −*e*^−*iθ*^*u*/∣*u*∣ + *v*/∣*v*∣ is an eigenvector of *Q*, with the eigenvalue *i* sin *θ*∣*u*∣∣*v*∣ (here, *θ* is the angle between *u*, *v*). This implies that the real and imaginary components of the eigenvector *w*_*k*_ = *p*_*k*_ + *iq*_*k*_ are obtained from the real and imaginary components *u*_*k*_ and *v*_*k*_ defining the matrix *A*^(*m*)^ via the linear transformation of the plane

$$\left(\begin{array}{c} p_{k}\\ q_{k} \end{array}\right)=\left(\begin{array}{c} {Lu}_{k}+{Mv}_{k}\\ {Nu}_{k} \end{array}\right)$$

where *L* = −cos *θ*/∣*u*∣, *M* = 1/∣*v*∣, and *N* = sin *θ*/∣*u*∣. Therefore, if *p*_*k*_ + *iq*_*k*_ are the components of the eigenvector corresponding to the rank 2 matrix stemming from a purely harmonic COOM with the offsets *α*_k_, *k* = 1, …, *n*, they are just obtained from the complex numbers *a*_*k*_ exp(*iα*_*k*_), *k* = 1, …, *n* by a linear transformation of the complex plane, and therefore, *the cyclic order defined by the arguments of these components* is the same as *the cyclic order of the collection of points on the unit circle* {cos(*α*_*k*_) + *i* sin(*α*_*k*_)}_*k*=1,…,*n*_.

Therefore, the spectral decomposition of the lead matrix can lead to the recovery of the order in which the signals are represented by the components of the time series **x**(**t**).

## ACKNOWLEDGMENTS

Yuliy Baryshnikov was partially supported by the ARO, through MURI SLICE. Fatima T. Husain was partially supported by a grant from DOD W81XWH-15-2-0032. Benjamin Zimmerman was partially supported by the National Institutes of Health grant R90AT008924-08. Data were provided [in part] by the Human Connectome Project, WU-Minn Consortium (Principal Investigators: David Van Essen and Kamil Ugurbil; 1U54MH091657) funded by the 16 NIH Institutes and Centers that support the NIH Blueprint for Neuroscience Research, and by the McDonnell Center for Systems Neuroscience at Washington University.

## SUPPORTING INFORMATION

A very early preprint was published on the *bioRxiv* preprint server: https://doi.org/10.1101/2021.05.16.444387. A supporting information to this manuscript provides a greater detail regarding the CA pipeline as well as the toolbox of persistent homology used here. Supporting information for this article is available at https://doi.org/10.1162/netn_a_00392.

## AUTHOR CONTRIBUTIONS

Ivan Abraham: Formal analysis; Investigation; Methodology; Software; Visualization; Writing – original draft. Somayeh Shahsavarani: Conceptualization; Validation; Writing – original draft; Writing – review & editing. Benjamin Zimmerman: Conceptualization; Validation; Writing – original draft; Writing – review & editing. Fatima Husain: Conceptualization; Validation; Writing – review & editing. Yuliy Baryshnikov: Conceptualization; Formal analysis; Methodology; Supervision; Writing – original draft; Writing – review & editing.

## FUNDING INFORMATION

Yuliy Baryshnikov, Army Research Office (https://dx.doi.org/10.13039/100000183), Award ID: MURI SLICE. Fatima Husain, U.S. Department of Defense (https://dx.doi.org/10.13039/100000005), Award ID: W81XWH-15-2-0032. Benjamin Zimmerman, Foundation for the National Institutes of Health (https://dx.doi.org/10.13039/100000009), Award ID: R90AT008924-08.

## DATA AVAILABILITY STATEMENT

The Human Connectome Project S1200 dataset is available for download from the project website with its own license agreement.

## Supplementary Material



## TECHNICAL TERMS

BOLD: Acronym for blood-oxygen level-dependent signal, a measure of oxygenation levels of different regions of the brain used as a proxy for neuronal activity.
Functional connectivity: Brain regions that show statsticially significantly correlated BOLD signals over a period of observation are said to be functionally connected.
Reparametrization invariant: Features of time series data that do not change with arbitrary monotonic transformations of the timeline are said to be reparametrization invariant.
Oriented area: The standard notion of area augmented with a ± sign depending on whether the orientation attached to the area’s enclosing boundary is counterclockwise or clockwise, respectiviely.
Lead matrix: A skew-symmetric matrix whose (*k*, *l*)-th entry records the oriented area computed between signals *x*_*k*_ and *x*_*l*_. The eigenvalues of this matrix are 0 or purely imaginary complex conjugate pairs.
Cyclic signals: Signals that repeat in time often absent any fixed or predictable period. Periodic signals are cyclic but cyclic signals need not be periodic. Analysis of such signals is called cyclicity analysis (CA).
Chain of offsets model: A model predicated on the assumption that the same unknown function *ϕ* generates different signals: *x*_*k*_ = *α*_*k*_*ϕ*(*t* − *α*_*k*_), possibly in the presence of corrupting noise.
Constellations: Each eigenvector of the lead matrix, with its complex valued entries considered as a collection of indexed points on the Argand plane, constitutes a constellation.
rs-fMRI: Time series data obtained from fluctuations of the BOLD signal in the brain in the absence of any cognitive or mental tasks, that is, at rest.
Persistence diagram: A scatterplot encoding the generation and annihilation of features (for the purposes of this paper, local maxima and minima) in a signal.
