# Supplementary material for: Hemodynamic cortical ripples through cyclicity analysis
Source: Netw Neurosci. 2024 Dec 10;8(4):1105–28. doi: 10.1162/netn_a_00392 (PMC11674492; doi:10.1162/netn_a_00392)
Supplement: Supplementary file 1 [file netn-8-4-1105-s001.pdf]

1. Cyclicity Analysis

1.1 Iterated integrals and universality

The problem of finding reparameterization invariants (RI) of trajectories

$$\mathbf{x}(t) = (x_1(t), x_2(t), \ldots, x_d(t))$$

in a multidimensional space $\mathbb{R}^d$ was addressed by [1], where the author established that essentially, all RI quantifiers are given by the *iterated integrals*, a family of functionals of trajectories that can be defined iteratively and computed efficiently. Chen's iterated integrals form a countable family: the degree of an iterated integral is one more than the number of iterative integrations needed to compute it. For each degree, there are several distinct (functionally independent) iterated integrals, and their number, and the overall structure are described by combinatorial apparatus relevant in several disjoint areas of mathematics. From empirical an perspective, however, the iterated integrals of higher orders become excessively prone to the noise, and we still don't have convincing examples of their practical usefulness.

1.2 Networks and Signals

Consider a weighted undirected graph G with n vertices, interpreted as a network, with edge lengths proportional to internode communication delays. Consider the following broadcasting protocol: if a node emits a signal, it goes to its neighbors, who will receive it after the edge-specific delay, and immediately rebroadcast it. If the signal was received by the node earlier, it ignores it. This implies that each signal reaches any given node along the shortest path connecting it to the source.

Such peer-to-peer propagation models are often referred to as *gossip, epidemic* or *first passage percolation* networks [2, 3], and are relevant in studies of social networks, peer-to-peer networks, distributed resource location, etc [4, 5, 6]. Assuming such a model, one can easily recover the structure of the underlying network, *if* the shortest path lengths between the pairs of the nodes are known. Suppose a signal $s_i(t) = f(t)$ propagates from a *source* node i. If the underlying graph G is a connected one, then a node $j \neq i$ of the network will eventually receive the signal from i as a delayed shapeform, so that

$$s_j(t) = f(t - d(i,j)) \tag{1}$$

where $d(i,j)$ is the shortest path distance between nodes i and j. For connected graphs, this distance is well-defined and finite even if there is no direct connection between nodes i and j. Quite often, one is only interested only in the ordinal information about the edgelengths (for example, the minimal spanning trees depend only on the ordering of the edge lengths). Can this ordinal information be reconstructed using *only* the observations of the (perhaps, noisy) signals at the nodes?

1.3 Analysis Pipeline

We will apply the CA pipeline to recover the network structure from the observations of the signals $s_k, k = 1, \ldots, n$, as it manifestly fits the *Chain Of Offsets Model* (see `Methods` section of the paper). For each of the $\binom{n}{2}$ pairs of signals given by (1) observed at the nodes of the network, we form the oriented area of the corresponding 2-dimensional projection as,

$$\text{area} = \frac{1}{2}\oint_C x\,dy - y\,dx = \frac{1}{2}\int_{t_1}^{t_2} x(s)y'(s) - y(s)x'(s)ds, \tag{2}$$

with $x, y = s_i, s_j$ for $i, j = 1, 2, \ldots, n$ and C being the parametric contour linearly closed up. As explained in the article, the orientation is indicated by attaching a sign to the computed area value, with a positive sign corresponding to counter-clockwise integration; interpreted as $j(t)$ *following* $i(t)$ in time.

The spectral decomposition of square matrix A whose entries are given by (2), i.e., the *lead matrix*, allows us to determine the relative distances of the nodes of the network to the seed node, where the signal originates. Indeed, as we argued previously, the order of the arguments of the components of the (complex) eigenvector corresponding to the leading (in absolute value) eigenvalue of the lead matrix will reflect the cyclic order of the propagating wave, if the rank two skew-symmetric lead matrix corresponding to the purely harmonic signal propagating through the network is a good enough approximation of the sampled lead matrix A. In this case, spectral analysis of the lead matrix recovers the lag-structure between the source node and every other node.

Note that one measure of how well the assumptions and results hold is obtained by computing the eigenvalue ratio $|\lambda_1(A)|/|\lambda_3(A)|$ of the matrix A, with larger ratios leading to better results. Repeating this analysis for various source nodes would then enable one to reconstruct the structure of the network.

1.4 Model example: Signal propagation in networks

To illustrate how the *Cyclicity Analysis* (CA) pipeline works, we introduce a model example, where the signals are propagating through a network by gossiping. This material is dedicated to the description of a simulated model, with which we illustrate the computational pipeline of tools we applied to the fMRI data.

Conceptually, this model, of the waves propagating in a network, broadcast from an unknown source, is a highly stylized caricature of the cortex waves. While we presented the results on the (tentative) recovery of those waves from the fMRI readings in the main text, it is enlightening to see what the situation is in a model example, where the ground truth is known.

1.5 Simulation study

In Figure 1 we introduce a network on $n = 12$ nodes.

The graph of Figure 1(a) is constructed by starting with the C_3 graph and adding nodes with $k = 3$ edges at a time. The edges are attached to vertices at random following a distribution proportional to the vertex degree [7]. The shown matrices represent the internode distances, both as given by the edge lengths in Figure 1(b), and by the shortest path distances between pairs of nodes in Figure 1(c). Note that the shortest distance using relays can be shorter than the direct link.

To generate the signal in Figure 1(d) emitted by the seed node (node 6 in this case), we use a convex combination of randomly modulated Gaussian functions, randomly displaced,

$$f(t) = \sum_{k=1}^{s} r_k g(t,k), \quad g(t,k) = \exp\left(-(t - k\pi + c\mathbf{R}_k)^2\right).$$

Here the displacements $\mathbf{R}_k$ and amplitudes r_k were drawn uniformly from the appropriate intervals (implementation of the simulation is available on a Github repository).

The signal propagates through the network according to the equation (1), to which we add a small noise, realized as the scaled Brownian motion, independently at each node.

1.6 Recovering the network structure

The results of the cyclicity processing pipeline for the time series generated in the example of Figure 1 are presented in Figure 2. Figure 2(a) shows the sampled lead matrix. The interpretation of the entries is quite intuitive; thus one can see, for example, the vanishing oriented area between 2nd and 4th nodes; this is consistent with the fact that the network distance from the *seed* node to either of them is approximately the same. The first pair of the conjugate eigenvalues dominates in the spectrum of the lead matrix, indicative of the high resolution of the analysis as shown in Figure 2(b). The eigenvector corresponding to (one of) the leading eigenvectors has components shown in Figure 2(c). The arguments of these components, that is the angles formed by the rays pointing towards them and the ray of positive real numbers are shown on the display Figure 2(d) as the scatter plot, against the shortest distance to the seed node. The strong, essentially linear dependence between these phases and the distances to the seed shows that cyclicity can be used to detect the latter from the former, in a reparameterization invariant way.

1.7 Areas' dynamics

Besides recovering the ordering of the signals represented by the time series, one can extract additional information from the oriented areas, as they are accumulated in time. Namely, one can consider the integrals (2) with a variable upper bound of integration, resulting in the functions $A_{ij}(t), i, j \leq n$:

$$A_{ij}(t) = \frac{1}{2}\int_0^t s_i(u)s_j'(u) - s_j(u)s_i'(u)\, du,$$

These functions give a richer characterization of the collective behavior of the network. As a visualization tool, we will use the area gain plots, as shown in Figure 3. Since $A_{ij} = -A_{ji}$ these plots are visualized in an upper triangular matrix, with a few of the plots magnified to emphasize some details. Some of the plots are flat-lines, which means there was no inferred *leader-follower* relationship between that particular pair of signals. Some plots show a sustained steady climb - *implying a strongly inferred leader-follower relationship*. The maximum gain (or drop) of the plots characterize the strength of the inferred directed relationship.The insets in the image show the combined time series of the select pairs of signals that generated them.

1.8 Robustness of dominant ROIs: statistical analysis

In approaching the results on the section on robustness of dominant ROIs, we choose as H_0 the assumption that the subsets are independent, not representing an underlying phenomena. Under such assumption, the probability that a random sample of size

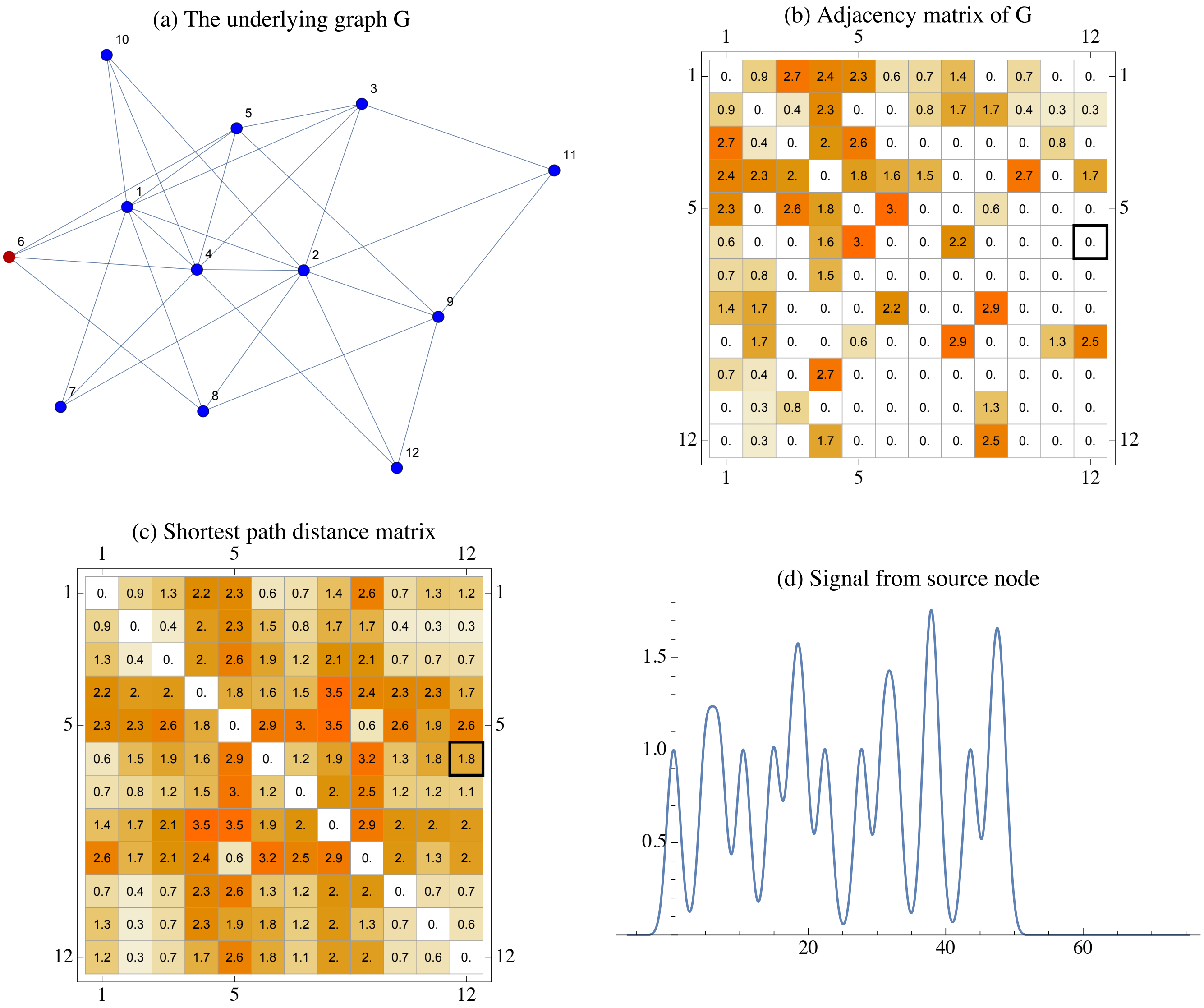

Figure 1. Details of the model example. **(a)** The graph G with $N = 12$ and source node $i = 6$, in red. **(b)** Random edge lengths. Zeros indicate the absence of a direct link between the nodes. **(c)** The shortest-path length matrix. Note that the connectivity of the graph results in all non-diagonal elements being nonzero. **(d)** The randomly generated the signal emanating from the source node.

k from the total set of N options is within the Levenshtein distance d from the given subset (i.e., the random subset has overlap of size at least $k-d$ with the given one) is

$$p(N,k,d) = \frac{\sum_{l=0}^{d} \binom{k}{l}\binom{N-k}{l}}{\binom{N}{k}}.$$

Specializing to our setting of $N = 68, k = 14$ and $d = 2$, we obtain

$$p(68,14,2) = 1.06284 \times 10^{-9},$$

leading to the conclusion that finding 12 dominant ROIs to be within the distance 2 from the one generated by the subset S essentially excludes the null-hypothesis.

1.9 (Non-)robustness with respect to reparametrization.

Lagged correlation methods for the detection of the directionality and speed of the propagation of the excitation through the brain are sensitive to the reparametrization of the timeline (unlike the CA, which, being dependent entirely on the iterated integrals, is manifestly invariant with respect to reparametrization). The following example exhibits a somewhat exaggerated

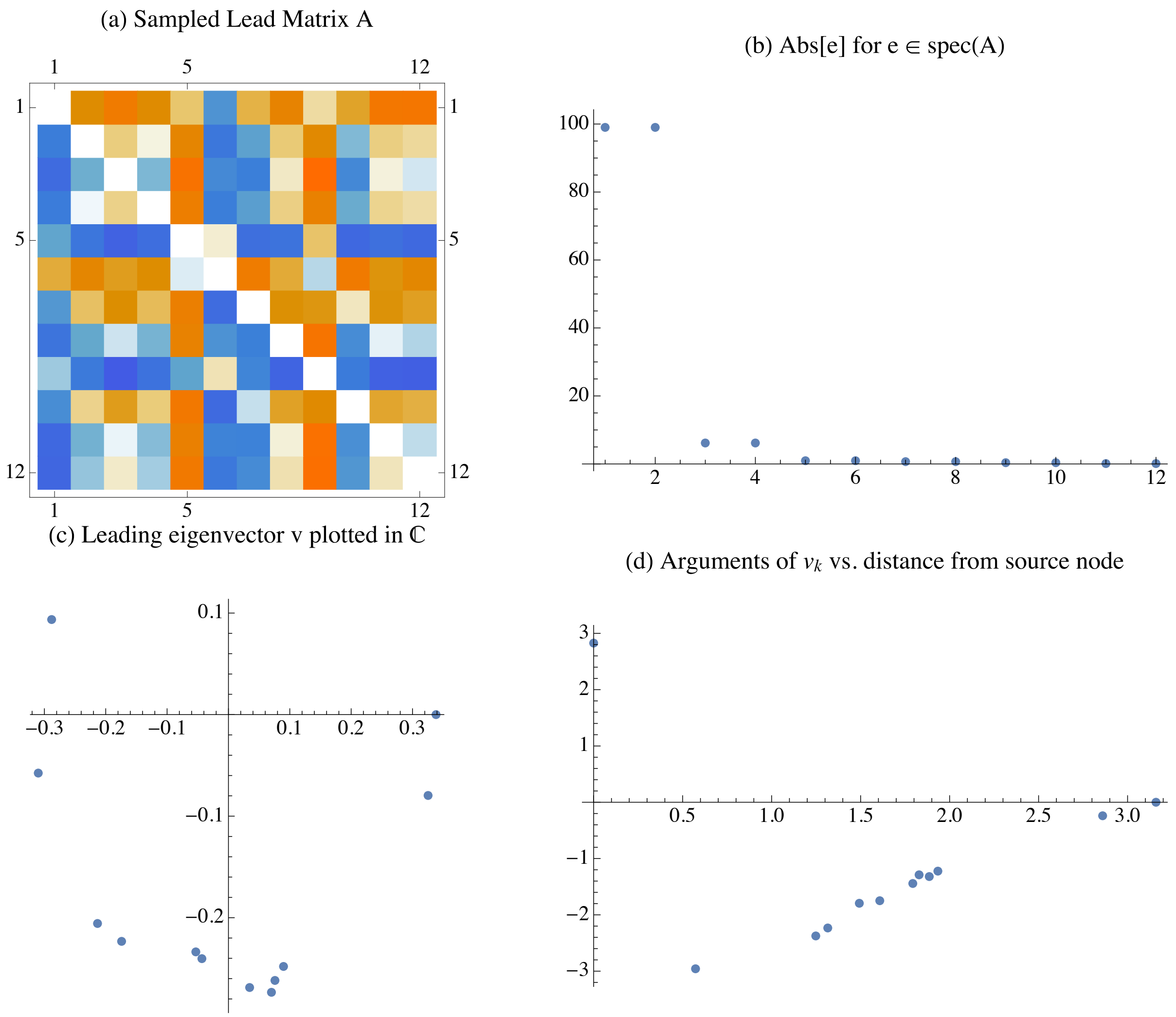

Figure 2. Results of CA for the model example. **(a)** The skew-symmetric lead matrix. **(b)** (Absolute values of) the eigenvalues of the lead matrix. The rank 2 matrix corresponding to the first two (complex-conjugated) eigenvectors approximates the lead matrix well, as the first pair of eigenvalues dominates: $|\lambda_1|/|\lambda_3| \approx 20$. **(c)** The "constellation" of the components of the leading eigenvector winds around the origin; the circular order of their arguments (phases) indicates the propagation of the gossip wave through the network. **(d)** More precisely, the scatter plot of these arguments against the *known* distances from the source node shows a near perfect linear dependence, implying that lag-structure is encoded with eigenvector. Typically the distances between the nodes and the source will be *unknown*, however repeated analysis with multiple sources and careful consideration of obtained complex arguments from the eigenvectors enables inference of the underlying pairwise relative distances.

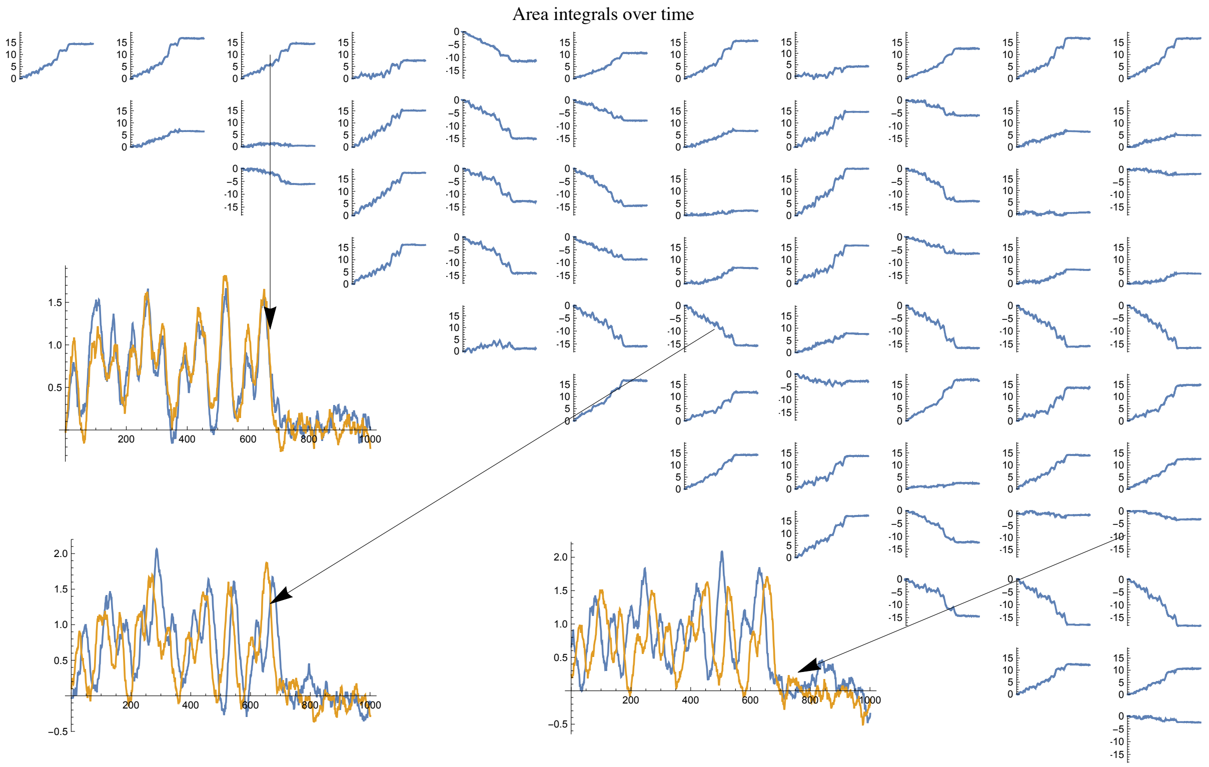

Figure 3. A panel showing the dynamics of the pairwise area-integrals computed as entries of the lead matrix. Since oriented areas are skew symmetric, the lower half shows insets with pairs of BOLD signals from ROIs that together generate the area dynamics. Shown are three typical instances: (top) strong directed relation as exhibited by increase, (bottom) strong directed relation as indicated by decrease and (right) a weak directed relationship as indicated my relatively flat line.

effect of such sensitivity. Namely, we consider three consecutive waves of a squared harmonic function, and its copy shifted by $1/4$ of the period (see the Figure 4 below).

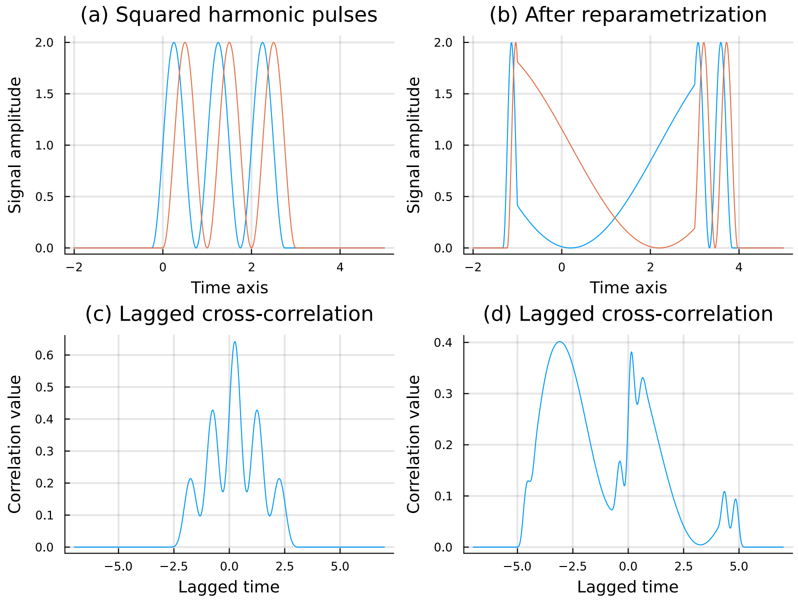

Figure 4. The top left plot shows a squared harmonic wave, with period 1 and its shift by $1/4$ of the period; the right is its reparametrization after a piece-wise linear time change. The bottom plots show the corresponding correlation functions (lagged correlations).

Applied to these two waveforms, the lagged correlation method returns, as expected 0.25 shift between the two waves. However, a simple reparametrization shown on the right display results in the imputed shift of -3.1: the imputed delay swaps the sign, so that now the orange series is deemed leading, and the blue one lagging. The underlying cross-covariance functions

$$c(\tau) = \int f(t)g(t-\tau)dt$$

are shown on the bottom display; the imputed lags correspond to the arguments where these functions attain their maxima.

2. Persistent Homology

We will describe here a small subset of the persistent homology theory, dealing with the basic case of 0-dimensional homology, - essentially, tracking the connecting components of the sublevel sets for a function f.

Consider a (continuous) function f of a topological space X (in our situation, the topological space is just a subinterval $X = [t_s, t_f]$ of the real line, but the definitions are valid in the generality we formulate them).

Consider a point $t_* \in X$ which is a *local minimum* of f: this means in some small vicinity $N_\varepsilon(t_*)$ (in our situation, again, the vicinity is just a subinterval $(t_* - \varepsilon, t_* + \varepsilon)$), the function does not dip below the level $f(t_*)$: $f(t) \geq f(t_*), t \in N_\varepsilon(t_*)$, and any path from t_* to outside of $N_\varepsilon(t_*)$ has to pass through a value that is strictly higher than $f(t_*)$.

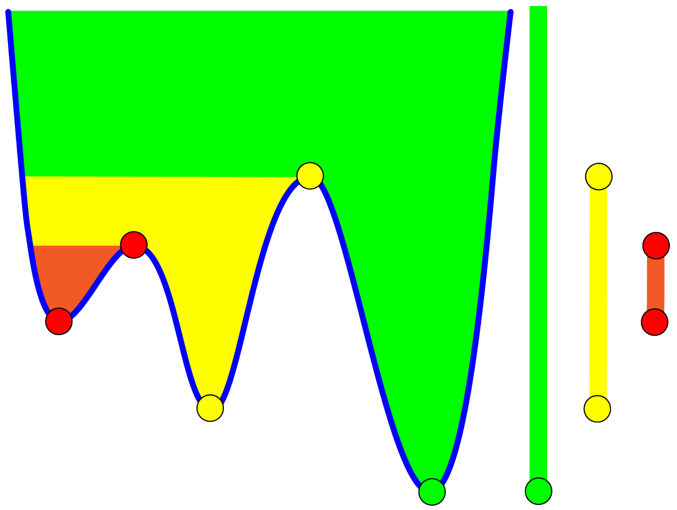

Figure 5. Filling the basin to determine the coupling of the local maxima and minima. The color coding signify the basins filled before a lower level can be reached. The corresponding persistence bars are shown on the right; the global minimum is coupled to $+\infty$.

Assume that the *global minimum* of f lies below $f(t_*)$. In this case, there is a path connecting t_* to a point where f attains a value below $f(t_*)$. What is the minimum of the maximal elevation $f(\cdot)$ along all such paths? It is easy to see that it is achieved at some point t^* with $f(t^*) > f(t_*)$ which is a saddle in the dimensions > 1, or a local maximum in the situation of $d = 1$, which is of primary interest to us. This point is well-defined in generic case (where the *critical values*, - the values of the function at its critical points, such as minima, saddles etc) are all different.

In this situation one says that the critical values $f(t_*)$ and $f(t^*)$ are *coupled*, and the pair $(b,d) = (f(t_*), f(t^*))$ forms a 0-*persistence bar*. The endpoints of this bar are referred as the *birth* and *death* values $b < d$, represented often as a point on the (b,d) plane. The collection of these points is referred to as the (0-dimensional) *persistence diagram*.

The *global minimum* is, by convention, always coupled to $+\infty$.

Informally, it is useful to think of the process of coupling of local minima t_* with their counterparts t^* as following: imagine f as the landscape that one fills with water, starting with a basin at t_*. Eventually, the water spills out to other basins, - the elevation at which it can be connecting to some *deeper* basin is coupled to the height of the local minimum t_*, see Figure 5 above.

Longer bars correspond to larger scale features of the function f, the intuition we use in the section discussing reversals in the oriented area signals in the main document.

3. Additional Figures

The number of dominant ROIs arising from the leading eigenvalue that were visualized in the main text was limited to either $k = 20$ or $k = 24$. The figures below show additional statistics for the case of $k = 28$ and $k = 54$. Figure 2 of the main text

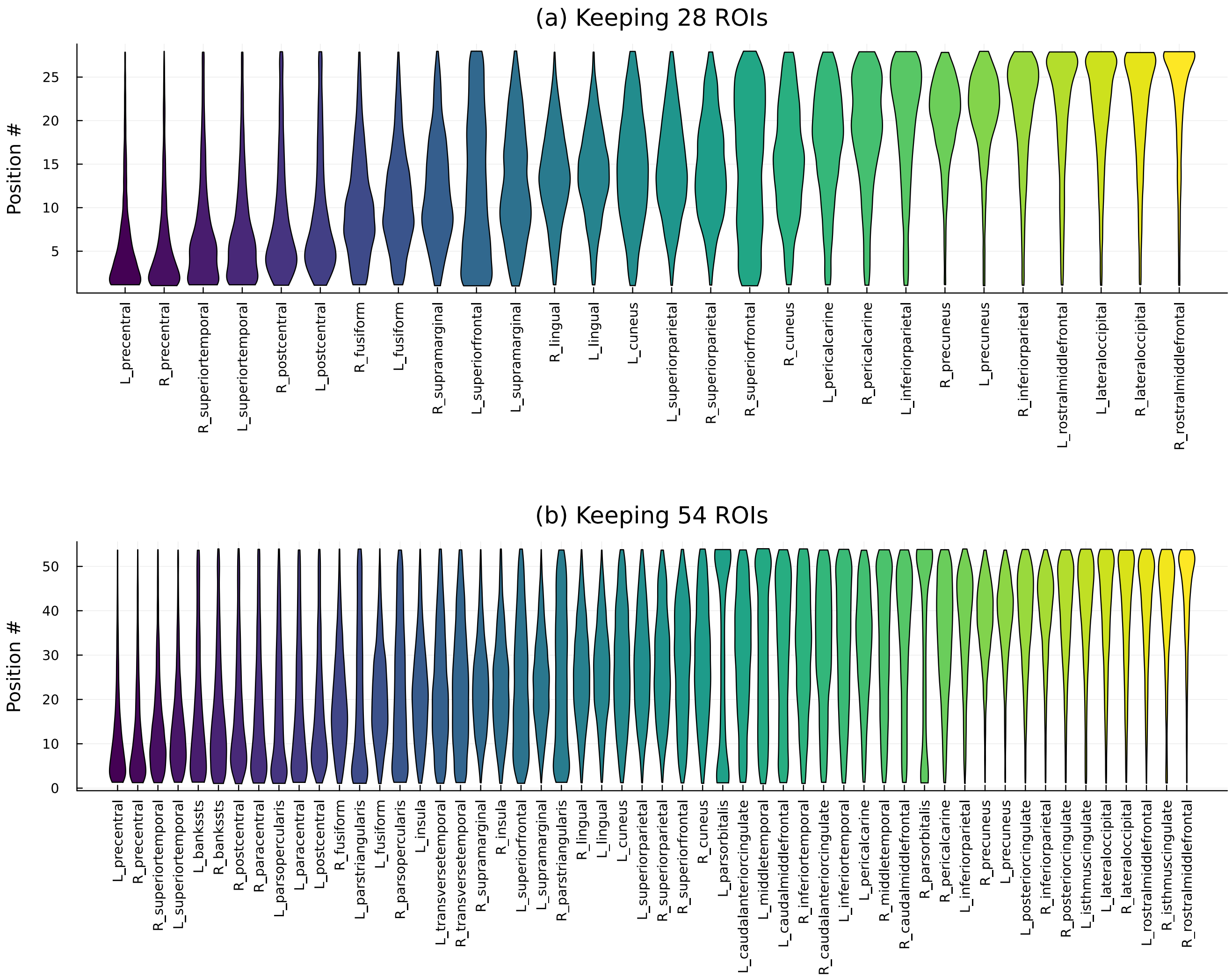

Figure 6. The above violin plot shows the distribution of the imputed ordering recovered when analysis is limited to $k = 28$ and $k = 54$ ROIs corresponding to the leading eigenvalue. One can see that in that in comparison to the plots from the main text, the ordering of the ROIs (obtained with lower values of k) remains unchanged even as more ROIs are included.

displayed the average rankings obtained for each ROI from the leading eigenpair based on the elliptic distance from the origin. The figure below shows the rankings obtained for the second most dominant eigenpair.

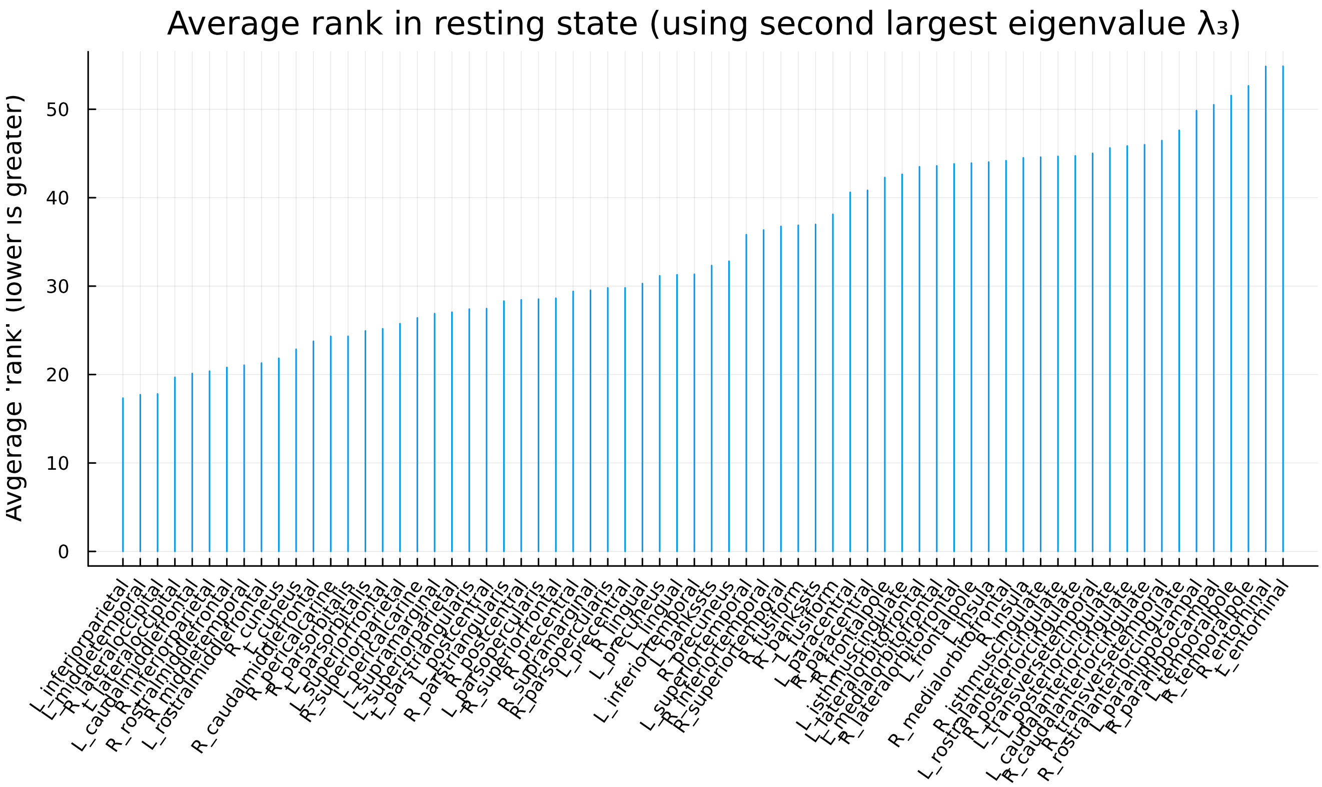

Figure 7. The above figure shows the ROIs ranked in terms of their elliptic distance for the second largest eigenvalue. The elliptic distance is a measure of how far an ROI is from the origin in its constellation (i.e. how dominating the signal is within the collection with respect to the first eigenvalue). The ranking depicted here is obtained by considering constellations from all resting state scans across all subjects and thus is the average rank.

References

[1] Kuo-Tsai Chen. Integration of Paths–A faithful representation of paths by noncommutative formal power series. *Transactions of the American Mathematical Society*, 89(2):395, Nov 1958.

[2] Antonio Auffinger, Michael Damron, and Jack Hanson. *50 years of first-passage percolation*. University lecture series. American Mathematical Society, Providence, RI, January 2018.

[3] Claudio Castellano, Santo Fortunato, and Vittorio Loreto. Statistical physics of social dynamics. *Reviews of Modern Physics*, 81(2):591–646, May 2009.

[4] Márk Jelasity, Alberto Montresor, and Ozalp Babaoglu. Gossip-based aggregation in large dynamic networks. *ACM Transactions on Computer Systems*, 23(3):219–252, Aug 2005.

[5] Indranil Gupta, Anne Marie Kermarrec, and Ayalvadi J. Ganesh. Efficient and adaptive epidemic-style protocols for reliable and scalable multicast. *IEEE Transactions on Parallel and Distributed Systems*, 17(7):593–605, 2006.

[6] João Leitão, José Pereira, and Luís Rodrigues. HyParView: A membership protocol for reliable gossip-based broadcast. In *Proceedings of the International Conference on Dependable Systems and Networks*, pages 419–428, 2007.

[7] Réka Albert and Albert László Barabási. Statistical mechanics of complex networks. *Reviews of Modern Physics*, 74(1):47–97, Jan 2002.
